# Analysis of miRNAs in Two Wheat Cultivars Infected With *Puccinia striiformis* f. sp. *tritici*

**DOI:** 10.3389/fpls.2019.01574

**Published:** 2020-01-10

**Authors:** Sowmya R. Ramachandran, Nicholas A. Mueth, Ping Zheng, Scot H. Hulbert

**Affiliations:** ^1^ Department of Plant Pathology, Washington State University, Pullman, WA, United States; ^2^ Department of Horticulture, Washington State University, Pullman, WA, United States

**Keywords:** microRNA, wheat (*Triticum aestivum*), stripe rust, *Puccinia striiformis*, cultivar-specific resistance

## Abstract

MicroRNAs are small RNAs that regulate gene expression in eukaryotes. In this study, we analyzed the small RNA profiles of two cultivars that exhibit different reactions to stripe rust infection: one susceptible, the other partially resistant. Using small RNA libraries prepared from the two wheat cultivars infected with stripe rust fungus (*Puccinia striiformis* f. sp. *tritici*), we identified 182 previously known miRNAs, 91 variants of known miRNAs, and 163 candidate novel wheat miRNAs. Known miRNA loci were usually copied in all three wheat sub-genomes, whereas novel miRNA loci were often specific to a single sub-genome. DESeq2 analysis of differentially expressed microRNAs revealed 23 miRNAs that exhibit cultivar-specific differences. TA078/miR399b showed cultivar-specific differential regulation in response to infection. Using different target prediction algorithms, 145 miRNAs were predicted to target wheat genes, while 69 miRNAs were predicted to target fungal genes. We also confirmed reciprocal expression of TA078/miR399b and tae-miR9664 and their target genes in different treatments, providing evidence for miRNA-mediated regulation during infection. Both known and novel miRNAs were predicted to target fungal genes, suggesting trans-kingdom regulation of gene expression. Overall, this study contributes to the current repository of wheat miRNAs and provides novel information on the yet-uncharacterized roles for miRNAs in the wheat-stripe rust pathosystem.

## Introduction

Small RNAs (sRNAs) are small, mobile noncoding RNA molecules that regulate gene expression and inhibit translation. Based on their biogenesis, small RNAs can be broadly classified into two major groups: microRNAs (miRNAs) and small interfering RNAs (siRNAs). MicroRNAs are 21–24 nt sequences encoded by plant microRNA genes (MIR) genes, which operate *via* post-transcriptional mRNA cleavage or translational inhibition (Kuan et al., 2016). In plants, miRNAs are processed from imperfectly complementary hairpin structures by the Dicer-like 1 (DCL1) endoribonuclease, which are then loaded into an RNA-induced silencing complex (RISC) to regulate expression of corresponding target genes. The RNA silencing mediated by microRNAs helps regulate myriad physiological processes such as plant growth, development, reproduction, and stress responses (Kulcheski et al., 2011; Parent et al., 2012; Qiu et al., 2016).

Recent advances in next-generation sequencing and availability of plant genomes have accelerated discovery of miRNAs and the processes under their regulation. With the availability of the wheat genome sequence, several groups have discovered wheat miRNAs that are conserved across plant species (Yao et al., 2007; Wei et al., 2009; Su et al., 2014; Achakzai et al., 2018) miRNAs involved in development (Li et al., 2013; Meng et al., 2013; Han et al., 2014; Li et al., 2015) and abiotic (Qiu et al., 2016) or biotic stress responses (Xin et al., 2010; Inal et al., 2014; Feng et al., 2015a; Feng et al., 2015b; Kumar et al., 2017). However, owing to the complexity of the wheat genome and the spatial/temporal nature of miRNA expression, a large number of miRNAs still remain to be discovered (Budak and Akpinar, 2015).

Wheat is a staple crop for more than 30% of the world population, with more than 220 million hectares under wheat cultivation worldwide. The crop is constantly subject to various abiotic and biotic stresses that limit crop production. Among these, stripe rust caused by *Puccinia striiformis* f. sp. *tritici* (Pst) constitutes a major problem for wheat cultivation, especially in cooler regions of the world under wheat production. The fungus can cause up to 100% reduction in yield on highly susceptible cultivars under favorable conditions, leading to severe crop losses (Chen, 2005). Control of the fungus has been mainly achieved through deployment of disease-resistant or partially resistant cultivars, which provide a safer and more economical alternative to the use of fungicides. Therefore, understanding the mechanisms of resistance becomes necessary for more effective control of the fungus. Although a few studies have looked at the expression of miRNAs in the wheat rust pathosystem and their role in resistance (Feng et al., 2015a; Feng et al., 2015b), much remains to be understood about the mechanisms underlying this complex interaction.

RNA silencing is a ubiquitous and fundamental process of gene regulation in eukaryotes, which controls several cellular processes including disease resistance. Recent studies have revealed differential accumulation of small RNAs when bacterial, fungal, and oomycete pathogens infect their hosts (Staiger et al., 2013; Yang L et al., 2013). At least five miRNAs: miR162 targeting DCL1 in rice, miR168 targeting AGO, miR818 targeting RDR2, miR1847 targeting AGO4, and miR873 targeting *Arabidopsis* chromomethylase 3, are upregulated during fungal infection (Baldrich et al., 2014; Ouyang et al., 2014; Baldrich et al., 2015; Zhang et al., 2015). Many defense-related processes like signal transduction leading to pathogen perception, oxidative stress response, hormone signaling, and antimicrobial enzymes have also been shown to be regulated by miRNAs (Xin et al., 2010; Radwan et al., 2011; Robert-seilaniantz et al., 2011; Zhang et al., 2011; Zhao et al., 2012.; Inal et al., 2014; Wong et al., 2014; Baldrich et al., 2015; Chávez-Hernández et al., 2015; Lunardon et al., 2016). In addition, intracellular nucleotide-binding domain leucine-rich repeat, or NLR proteins, are another important class of pathogen defense genes controlled by pathogen-responsive miRNAs (Fei et al., 2015). Many NLR genes are targets of the miR482 superfamily in tomato (Shivaprasad et al., 2012) and miR2118 in *Medicago truncatula* (Zhai et al., 2011). Upon infection with viruses and *Pseudomonas syringae* DC3000, levels of miR482 were reduced, with a consequent increase in NBS-LRR genes and resistance to disease (Shivaprasad et al., 2012). These genes also serve as phasiRNA-generating loci that produce secondary siRNAs involved in suppression of many other NLR genes (Zhai et al., 2011; Li et al., 2012; Fei et al., 2013; Fei et al., 2015).

Previous reports have demonstrated rapid and specific small RNA induction contributing to host defense (Katiyar-Agarwal and Jin, 2010; Zhang et al., 2011; Swaminathan et al., 2013). Although miRNAs have been known to function in plant immunity, it is unknown whether various plant cultivars differentially express defense-related miRNAs that could be manipulated in breeding programs. Here we report the identification of 163 novel miRNAs and 182 known miRNAs from two cultivars of wheat under stripe rust infection. Twenty-three of these miRNAs were differentially expressed under stripe rust infection in a cultivar- or pathogen-specific manner. Further, a target for miRNA tae-miR9664 was confirmed using 5’ RNA Ligase-Mediated Rapid Amplification of cDNA Ends (5’RLM-RACE), and the corresponding target was found to be differentially expressed between the two cultivars tested.

## Material and Methods

### sRNA Library Preparation and Sequence Analysis

This study used small RNA libraries generated by Mueth et al., 2015 (NCBI BioProject Acc. No. PRJNA289147). Two wheat varieties, “Louise” (exhibiting partial resistant to stripe rust) and “Penawawa” (susceptible to stripe rust), were grown in the greenhouse at 25°C for 16 h light; 15°C for 8 h dark. Six-week-old plants (Feekes growth stage 9) were inoculated with *P. striiformis* spores (race PST-100) and placed in the dew chamber for 24 h. Then, plants were transferred to a climate-controlled growth chamber for three additional days (16°C for 16 h light; 8°C for 8 h dark). Flag leaves were excised, flash-frozen, and ground, then small RNA was purified using the mirVana miRNA Isolation Kit (Thermo Fisher Scientific, USA). Small RNA libraries were constructed and sequenced using the Ion Proton platform (Life Technologies, USA) at the Washington State University Genomics Core. Adaptors and barcodes were trimmed after assigning sequences to respective libraries, and sequences were filtered to remove low quality reads (PHRED <15) using Ion Torrent software.

Small RNA reads from different libraries were pre-processed using CLC Genomics Workbench versions 7 and 8 (QIAGEN, Aarhus, Denmark). Sequences 17-34 nucleotides in length with ≥ 10 reads (pooled from all libraries) were input to miRDeep-P software (Yang and Li, 2011) for miRNA prediction. Mapping in miRDeep-P was performed using the Chinese Spring wheat genome sequence provided by IWGSC (http://www.wheatgenome.org/). Multi-mapped reads were filtered to include reads mapping to ≤100 locations in the wheat genome. MicroRNAs predicted by miRDeep-P were further processed to remove non-coding RNAs (such as rRNAs and tRNAs) by filtering out reads that perfectly matched sequences in the Rfam database (http://rfam.xfam.org/), as well as reads perfectly matching the wheat repetitive elements database (http://www.girinst.org/repbase/update/search.php). Predicted and filtered miRNAs were then classified into known and novel miRNAs using the “annotate and merge” function of CLC Genomics Workbench small RNA pipeline. The 119 sequence entries for wheat miRNAs from miRBase (Release 22.1, http://www.mirbase.org) were used for searching known miRNAs. Additional wheat miRNA sequences not available on miRBase were compiled from literature (Yao et al., 2007; Wei et al., 2009; Xin et al., 2010; Li et al., 2013; Feng et al., 2015a; Feng et al., 2015b). miRBase was also searched to identify novel miRNAs that match known miRNAs from other plant species. The novel miRNAs names were given the prefix “tae-miR” based on miRBase convention. miRNA identifiers following the prefix are unique names that are not previously reported in miRBase or other studies reporting miRNAs from wheat. Annotation of orthologs and paralogs were based on sequence similarity to mature miRNA sequences. Known miRNA variants were named using suffix a/b, etc. to denote sequence variation from existing miRNA sequence.

Genome-mapped reads were used to perform differential gene expression analysis in DESeq2 (https://bioconductor.org/packages/release/bioc/html/DeSeq2.html). DESeq2 performs normalization of raw read counts using median ratio normalization. Both normalized counts and log_2_ fold change values were obtained using DESeq2. miRNAs were called differentially expressed between treatments if the log_2_ fold change difference was ≥ 1.5 or ≤ -1.5 and statistically significant at a False Discovery Rate of 5% using the Benjamini-Hochberg procedure in R.

### Real-Time-Quantitative PCR (RT-qPCR)for sRNA and Target Genes

A fraction of the RNA samples used for sequencing was used for RT-qPCR as described by Ro and Yan (2010). Small RNA was polyadenylated using Poly(A) polymerase (NEB, USA) and reverse transcribed using a long miRTQ primer. Quantitative PCR was performed using an RNA-specific primer and the miRTQ primer at 95°C for 3 min, 35 cycles at 95°C for 15 s, 60°C for 45 s on MyiQ Single-Color Real-Time PCR detection system (BioRad, USA). A wheat U6 primer was used as a control for calculating fold change. Relative gene expression was calculated using the 2^‑ΔΔCT^ approach (Livak and Schmittgen, 2001) using three biological replicates.

Transcript levels of target genes were quantitated using total RNA from flag leaves. cDNA was synthesized using iScript reverse transcription Supermix for RT-qPCR (Bio-Rad, USA) and real-time quantitative PCR was performed using iTaq Universal SYBR Green Supermix (Bio-Rad, USA). PCR was performed on the MyiQ Single Color Real-Time PCR detection system (Bio-Rad) under the following conditions: 95°C for 3 min, 40 cycles of 95°C for 15 s, 60°C for 45 s; followed by a dissociation program to obtain the melting curves. The wheat *GAPDH* gene was used as an internal reference control for relative gene expression calculation using three biological replicates. Two independent inoculations were performed for RT-qPCRs to analyze target gene and the mature miRNA.

### Target Analysis and 5’ RACE

MicroRNA target prediction was performed using psRNATarget (http://plantgrn.noble.org/psRNATarget/) and TAPIR (https://omictools.com/tapir-tool) using the Washington Wheat Transcriptome (provided by the Western Regional Small Grains Genotyping Lab) and *P. striiformis* PST-78 transcriptome (https://www.broadinstitute.org/scientific-community/science/projects/fungal-genome-initiative/puccinia-comparative-genomic-projects). Schema V1 (2011 release) settings were used for psRNATarget with an expectation value of 3.0. An expectation value of 3.0 was also used for TAPIR. Results from the two programs were pooled to obtain a list of putative targets for both *P. striiformis* and wheat. The resulting lists of targets from TAPIR and psRNATarget were scanned for protein signatures using InterProScan followed by functional annotation using Blast2Go version 3.2 (https://www.blast2go.com) for assigning gene ontology (GO) terms. Protein sequences of putative *Puccinia* targets were retrieved from the BROAD Institute *P. striiformis* PST-78 protein sequences at the *Puccinia* comparative genomics projects (ftp://ftp.broadinstitute.org/pub/annotation/fungi/puccinia/genomes/puccinia_striiformis_pst-78/). Signal peptide prediction was done using Phobius (phobius.sbc.su.se/) on default settings.

RNA ligase-mediated 5’ RACE was performed to confirm the target for miR399b, as described by First Choice™ RLM-RACE kit (Invitrogen). Total RNA was isolated from infected and uninfected flag leaves using TRIzol reagent and enriched for poly(A) mRNA using the Oligotex Direct mRNA kit (QIAGEN, USA). Ligation of the RNA oligonucleotide (250 ng) and poly(A) RNA (100 ng) was carried out in the presence of T4 RNA ligase; ligated RNA was reverse transcribed using Superscript III (Invitrogen, USA). The resulting cDNA was used for PCR amplification of degraded target genes. PCR products of the expected size were gel-eluted and cloned into the pGEM T-Easy vector (Promega, USA). Plasmid DNA was isolated from at least 10 positive clones (confirmed by colony PCR) and sequenced at ELIM Biopharm (USA) to map the cleavage site of the predicted target.

## Results and Discussion

### Sequencing and Mapping

To assess the involvement of small RNAs in stripe rust infection, we profiled miRNA accumulation from two cultivars of wheat. Variety “Penawawa” is susceptible to stripe rust, while variety “Louise” has partial high-temperature adult plant resistance (HTAP) *via* a locus on chromosome 2BS (Carter et al., 2009). Louise exhibits an infection type (IT) of 2–3 while Penawawa has a rating of 6–8 when rated based on a 0–9 scale (Carter et al., 2009). Despite the widespread use of HTAP resistance in the field, little is known about its mechanism for disease resistance. Thus, this study analyzes cultivar-based miRNA profiles between compatible and partially incompatible interactions to elucidate the potential role of miRNAs in stripe rust infection. An intermediate time point (4 days post infection) was chosen for analysis to capture gene expressional changes associated with disease after the fungus has colonized the plants and is expanding through the host tissue. Small RNAs were divided into four treatment groups: infected Penawawa (IP), healthy Penawawa (HP), infected Louise (IL), and healthy Louise (HL).

**Figure 1:**
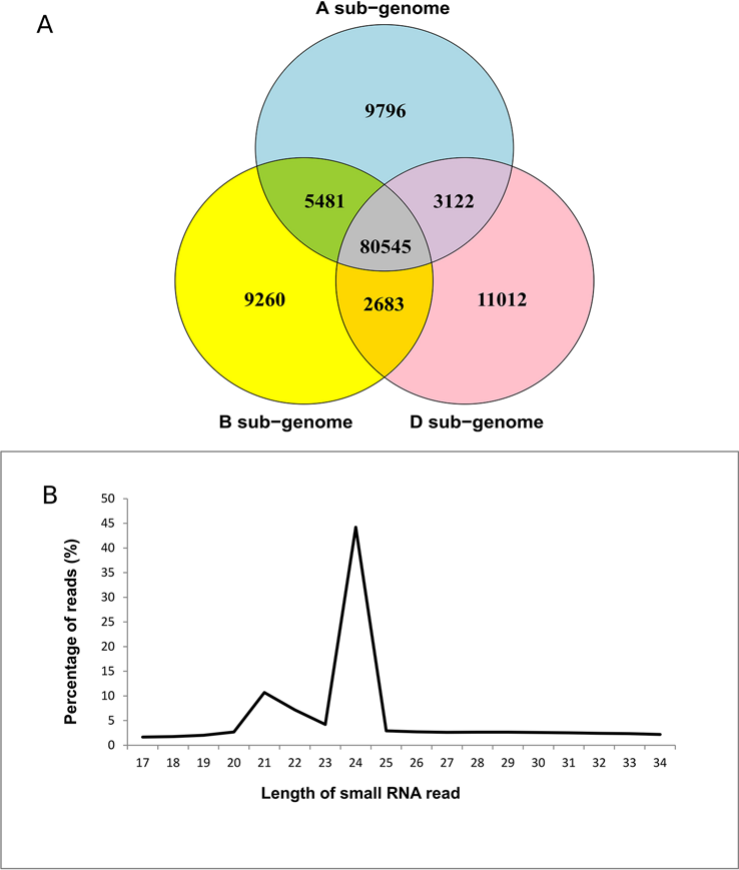
Summary of small RNAs that map to the wheat genome. **(A)** Distribution of mapped reads in the three sub-genomes of wheat. **(B)** Length distribution of reads mapping to the wheat genome.

Small RNAs 17–34 nt in length were compared to obtain a set of non-redundant (unique) sequences (**Supplementary Table 1**). The dataset was enriched for wheat-specific sRNAs by mapping non-redundant sequences to the wheat Chinese Spring draft genome. A total of 67% sequences mapped to the wheat genome. The proportion of unmapped reads could be attributed to an incomplete wheat genome or DNA sequence polymorphisms between the two cultivars used in this study and the reference genome. A similar trend was also observed for rice sRNAs (Li Y et al., 2014). Of the mapped sequences, 98,944, 97,969, and 97,362 mapped to the A, B, and D sub-genomes of wheat, respectively, making up approximately 80% of the total (**Table 1**, **Figure 1A**). These results were consistent with previous reports on wheat small RNAs.

**Table 1:** Summary of unique sRNA sequences mapping to the wheat genome.

Small RNAs mapped to the wheat genome	Sequences	Percentage
Total unique sequences	182,071	
Sequences mapping to wheat genome	121,899	67
**Sequences mapped to:**		
A sub-genome	98,944	81
B sub-genome	97,969	80
D sub-genome	97,362	80
Sequences common to ABD sub-genomes	80,545	66

The 20–24 nt size range accounted for more than 66% of total reads, suggesting DCL-mediated processing of sRNAs (**Figure 1B**). Approximately 40% of sRNA reads were 24 nt in length (**Figure 1B**, **Supplementary Table 2**). This accords with previous findings, where 24 nt sRNAs accounted for a majority (Meng et al., 2013; Han et al., 2014; Pandey et al., 2014; Li et al., 2015). Since 24 nt siRNAs commonly guide chromatin remodeling, the predominance of this size sRNAs suggests regulation of gene expression *via* heterochromatin formation in wheat (Allen and Howell, 2010; Law and Jacobsen, 2010; Pandey et al., 2014). Other read lengths based on abundance were 20 nt (2.7%), 21 nt (10.7%), 22 nt (7.2%), and 23 nt (4.2%) (**Figure 1B**). Previous studies have also shown accumulation of 21, 22, and 23 nt small RNAs in both monocot cereals and dicots like cotton and tomato that are involved in chromatin remodeling (Moxon et al., 2008; Yang X et al., 2013; Pandey et al., 2014). Furthermore, 66% of sRNAs mapped to all three sub-genomes of wheat, while 24.7% mapped exclusively to either the A, B, or D sub-genomes (**Figure 1A**). Gardiner et al. (2015) previously observed a sub-genome specific methylation pattern that correlated with the differential gene expression in the three wheat sub-genomes. It is possible the small percentage of reads originating exclusively from the three sub-genomes contribute towards sub-genome-specific methylation of the A, B, and D genomes (Li A et al., 2014; Gardiner et al., 2015). We also observed a smaller proportion of 24 nt sRNAs in infected Penawawa vs. infected Louise plants (*p* < 0.001, *z-*test on proportions, **Supplementary Table 3**). This suggests that fungal infection may lead to global changes in small RNA expression as previously noted for powdery mildew-wheat pathosystem (Xin et al., 2010).

### Known and Novel Wheat MiRNAs

After mapping the reads to the wheat genome, unique sRNAs were processed using miRDeep-P to identify plant microRNAs. In total, we identified 182 miRNAs with sequences identical to known miRNAs (**Supplementary Tables 4** and **5**). Of these, 119 matched sequences in miRBase, while 63 matched sequences reported from previous studies not yet listed in miRBase (**Table 2**). Variants contained one or two mismatches to miRNAs in miRBase and previously discovered miRNAs.

**Table 2:** Summary of known miRNAs.

Annotation	Number of miRNAs
mirBase	119
Other wheat miRNA studies	63
**Variants Total**	119
In mirBase	85
In other wheat miRNA studies	34
Conservation of known miRNAs in other plants	
Dicots	36
Monocot-specific	79

When assessed for conservation in different plant genera, 36 of the 182 known miRNAs identified in this study were conserved between monocots and dicots, while 79 were monocot-specific (**Table 2**). The top three most abundantly expressed miRNAs in our dataset were tae-miR9772, tae-miR159c, and tae-miR9674b. Additionally, many miRNAs identified by Feng et al., 2015b (mostly variants of miRNAs from other plant species) were some of the most highly expressed miRNAs in our study. These were ata-miR166a-3p, ata-miR172b-3p, ata-miR393-5p, and hvu-miR168-5p. Other Pooideae-specific miRNAs like tae-miR5200 was also among the most highly expressed.

**Figure 2:**
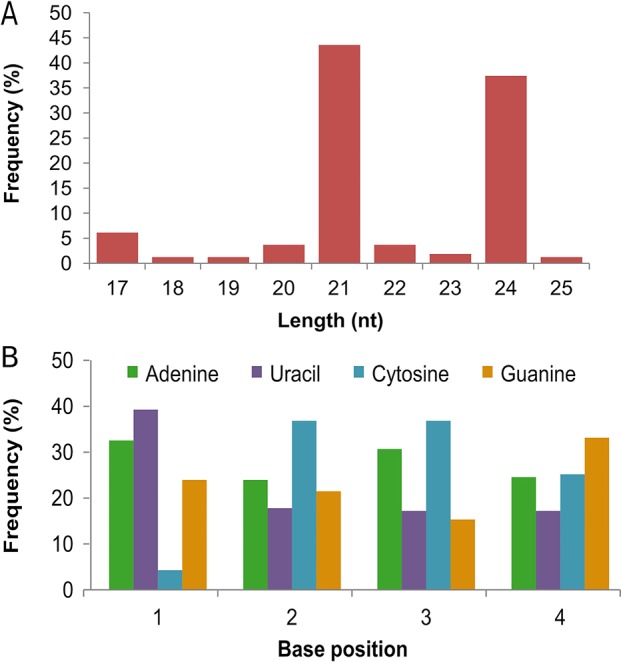
Characteristics of novel miRNAs. **(A)** Size distribution of novel miRNAs. **(B)** Frequency distribution of first four nucleotides in the identified novel miRNAs.

Several small RNA sequencing efforts have helped in identifying conserved and non-conserved microRNAs in wheat (Yao et al., 2007; Wei et al., 2009; Xin et al., 2010; Li et al., 2013; Pandey et al., 2014; Feng et al., 2015a; Feng et al., 2015b). However, owing to little overlap between non-conserved miRNAs between different studies and their condition-specific expression, many miRNAs still remain to be discovered. Here, we identified 163 novel miRNAs based on the secondary structures of their precursor RNA (**Table 3**, **Supplementary Tables 6A** and **6B**). Most of these were 21 nt (71 miRNAs) or 24 nt (61 miRNAs) in length (**Figure 2A**). Most novel miRNAs exhibited a strong preference for uracil or adenine at their 5’ position with least preference for cytosine (**Figure 2B**), which is consistent with previous reports (Su et al., 2014; Sun et al., 2014; Feng et al., 2015a). Among the 163 novel miRNAs, 48 (29%) had 15 or more reads in at least one of the four treatments. In addition, 15 novel miRNAs were detected only in Louise, and 7 miRNAs were detected only in Penawawa. While some of these cultivar-specific miRNAs were not very abundant, tae-miR107, tae-miR118, and tae-miR33 averaged more than 10 reads in Louise and none in Penawawa (**Table 3**, **Supplementary Table 6A**).

**Table 3:** Top 10 highly expressed novel (not previously reported, see *Materials and Methods* for details) miRNAs identified in this study and their corresponding expression levels in four different treatments. Numbers represent average reads per million of three replicates.

miRNA Name	Sequence (5’ to 3’)	IL	HL	IP	HP
tae-miR143	TGAAGAGCGCGGGCAGCACAA	265.7	263.9	192.4	307.1
tae-miR92	GGGCTATTAGCTCAGTGGTA	136.9	185.9	116.5	192.4
tae-miR117	TCCGAGTGGCGGCACCA	135.8	163.8	150.2	153.3
tae-miR132	TCCTACTTGGGGAGCCA	130.6	128.6	200.5	212.9
tae-miR95	GTCCCAGCGTGGTCGCCA	122.8	112.2	131.0	128.6
tae-miR34	AGCCAACAACCTCCTAGTTCC	119.8	80.9	77.0	124.1
tae-miR36	AGCGGAGTAGAGCAGT	110.2	225.4	149.6	167.1
tae-miR102	TCACAAATATAAGATGTTCTG	102.3	115.0	116.2	120.4
tae-miR107	TCATGGCAGCAACCGGGACTA	87.4	83.6	0.0	0.0
tae-miR133	TCCTGCCGACCACGCCA	80.7	77.3	78.6	77.5

IL, Infected Louise; HL, Healthy Louise; IP, Infected Penawawa; HP, Healthy Penawawa.

Next, we searched for conservation of novel miRNAs in other plant species. Of 163 miRNAs identified, 10 were variants (≤2 mismatches) of miRNAs from other monocots—*Aegilops tauschii*, *Brachypodium distachyon, Hordeum vulgare, Festuca arundinacea, Oryza sativa*, and *Zea mays*, while 7 matched miRNAs from dicot species—*Citrus sinensis, Populus trichocarpa, Theobroma cacao,* and *Medicago truncatula* (**Supplementary Table 7**). We found tae-miR43, tae-miR122, and taemiR-155 to be variants of taemiR818a, taemiR6220, and taemiR3633, respectively (Achakzai et al., 2018). The lack of homology with known miRNAs suggests that the majority of novel miRNAs identified are not conserved across diverse plant families. A similar trend has been reported previously in wheat (Pandey et al., 2014) and other monocots (Mondal et al., 2015). In plants, a minority of miRNA genes are evolutionarily conserved, while a majority of species-specific miRNA precursors are recent in origin (Fahlgren et al., 2007; Cuperus et al., 2011). The presence of a large proportion of wheat-specific miRNAs provides further proof for the rapid and independent evolution of a diverse subset of miRNAs in each species (Lu et al., 2008).

**Figure 3:**
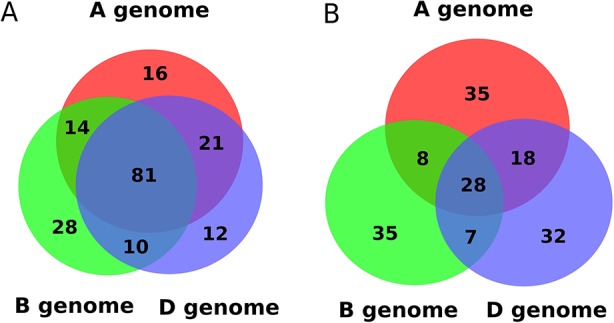
Distribution of: **(A)** known and **(B)** novel miRNAs in the three sub-genomes of wheat.

We compared the genomic locations of predicted miRNA precursors, finding that 44.5% of known miRNAs have one or more homologs in all three sub-genomes of wheat (**Figure 3A**). In contrast, only 17.1% of novel miRNAs had one or more homologs in all three wheat sub-genomes, and most originated from a single sub-genome (**Figure 3B**). If a microRNA locus originated in a common ancestor of the progenitors of hexaploid wheat, then each sub-genome is likely to carry an orthologous version. Conversely, a microRNA precursor that appears exclusive to one sub-genome may be evolutionarily more recent.

### Differentially Expressed miRNAs During Stripe Rust Infection and in Two Cultivars of Wheat

MicroRNAs regulate gene expression under abiotic and biotic stress. To investigate whether the identified miRNAs are differentially expressed between the two cultivars, and during stripe rust infection, we profiled for differential expression using DESeq2. MicroRNA expression levels were compared four different ways: IL vs. HL, IP vs. HP, HL vs. HP, and IL vs. IP. Fold changes in expression were calculated along with their corresponding probabilities.

Twenty-three miRNAs were differentially expressed between the four different treatments. TA078/miR399b was significantly different between healthy and infected plants of both cultivars. This miRNA was expressed 1.7-fold higher in infected Penawawa when compared with healthy Penawawa. Meanwhile, it was expressed 1.9-fold lower in infected Louise than in healthy Louise. An induction in miR399 expression was also observed in citrus trees infected with Huanglongbing and conditions of phosphate starvation (Zhao et al., 2013). Unlike studies comparing highly resistant and susceptible cultivars, we did not observe a huge shift in miRNA expression profile during infection. This may be due to the comparison of a partially resistant and susceptible cultivar that does not differ much in their reaction to stripe rust (Inal et al., 2014). However, it is interesting to note that miRNA-mediated gene regulation may also be involved in quantitative resistance to stripe rust, which is not yet reported in literature.

A total of 23 miRNAs were differentially expressed between Penawawa and Louise (**Table 4** and **Supplementary Table 8**). Of these, 19 miRNAs were differentially expressed between healthy Penawawa vs. healthy Louise, 16 showed differences between infected Penawawa vs. infected Louise, while 12 were common to both comparisons. Since cultivar-based differences for three miRNAs (tae-miR399a, tae-miR9666b, and Ta-miR2027a) were apparent only during infection, these miRNAs and their corresponding targets may be involved in resistance to stripe rust. Reads for three miRNAs, tae-miR107, tae-miR118, and tae-miR1123a, were only detected in Louise across infected and healthy treatments suggesting a cultivar-specific difference in miRNA expression.

**Table 4:** Differentially expressed microRNAs.

miRNA name	IL/HL			IP/HP			IP/IL			HP/HL		
	Fold diff	*p*-value	FDR Correction	Fold diff	*p*-value	FDR Correction	Fold diff	*p*-value	FDR Correction	Fold diff	*p*-value	FDR Correction
tae-miR1123a	–0.4	0.57	1.00	0.0	0.57	1.00	–4.4	0.00	0.00	–5.2	0.00	0.00
tae-miR397a	0.9	0.03	1.00	0.5	0.03	1.00	–2.1	0.00	0.00	–1.7	0.00	0.00
tae-miR399a	–1.1	0.03	1.00	1.5	0.03	1.00	2.4	0.00	0.00	–0.1	0.83	1.00
tae-miR5049b	0.0	1.00	1.00	0.2	1.00	1.00	3.2	0.00	0.03	3.2	0.00	0.02
tae-miR531a	0.0	0.97	1.00	0.0	0.97	1.00	–6.1	0.00	0.00	–6.5	0.00	0.00
tae-miR9664	–1.0	0.02	1.00	0.3	0.02	1.00	–2.0	0.00	0.00	–3.3	0.00	0.00
tae-miR9666b	0.6	0.32	1.00	1.4	0.32	1.00	2.3	0.00	0.00	1.6	0.01	0.12
PC-3p-65443_14a	0.0	1.00	1.00	0.2	1.00	1.00	4.3	0.00	0.00	4.4	0.00	0.00
PC-5p-32883_33a	0.2	0.87	1.00	0.0	0.87	1.00	–2.9	0.01	0.08	–3.0	0.00	0.04
Ta-miR2027a	0.9	0.34	1.00	0.0	0.34	1.00	–3.4	0.00	0.02	–2.8	0.00	0.05
TA078/miR399b	–1.9	0.00	0.04	1.7	0.00	0.04	2.5	0.00	0.00	–1.1	0.02	0.18
tae-miR2	–0.5	0.62	1.00	0.0	0.62	1.00	–2.5	0.01	0.14	–3.3	0.00	0.02
tae-miR100	0.0	1.00	1.00	–0.6	1.00	1.00	3.1	0.00	0.02	4.0	0.00	0.00
tae-miR107	0.1	0.87	1.00	0.0	0.87	1.00	–6.5	0.00	0.00	–6.8	0.00	0.00
tae-miR118	–0.5	0.44	1.00	0.0	0.44	1.00	–4.6	0.00	0.00	–5.5	0.00	0.00
tae-miR134	–0.8	0.50	1.00	0.0	0.50	1.00	–2.1	0.04	0.27	–3.1	0.00	0.04
tae-miR15	–0.2	0.88	1.00	0.0	0.88	1.00	–2.6	0.01	0.12	–3.1	0.00	0.04
tae-miR200	–1.2	0.07	1.00	–0.9	0.07	1.00	3.7	0.00	0.00	3.4	0.00	0.00
tae-miR206	0.0	0.96	1.00	0.0	0.96	1.00	–3.0	0.00	0.05	–3.3	0.00	0.02
tae-miR33	0.4	0.57	1.00	0.0	0.57	1.00	–4.3	0.00	0.00	–4.3	0.00	0.00
tae-miR57	0.4	0.43	1.00	–0.6	0.43	1.00	0.8	0.17	0.60	1.8	0.00	0.02
tae-miR65	0.0	1.00	1.00	–0.2	1.00	1.00	3.9	0.00	0.00	4.4	0.00	0.00
tae-miR96	0.1	0.87	1.00	0.0	0.87	1.00	–2.9	0.00	0.07	–3.1	0.00	0.03

IL, Infected Louise; HL, Healthy Louise, IP, Infected Penawawa; HP, Healthy Penawawa.

### Known miRNA Target Prediction and Validation

Targets for known and novel miRNAs were predicted using the *T. aestivum* and *P. striiformis* transcriptomes as references. A hundred and seventy-nine of the known miRNAs including variants targeted at least one wheat gene, while 140 novel miRNAs were predicted to target wheat transcripts (**Supplementary Tables 9** and **10**). Wheat targets included a large number of genes involved in regulation of transcription and associated with the GO term ADP binding, including NBS-LRR genes. It was interesting to note that 51 out of 179 known miRNAs with targets had at least one target annotated as disease resistance-like protein or NBS-LRR protein (**Supplementary Table 9**). Among these, tae-miR1123a, tae-miR5049b, tae-miR9664, and PC-5p-32883_33a were also differentially expressed in at least one of the four treatment comparisons, suggesting their involvement in stripe rust infection. Forty-two novel miRNAs also targeted disease resistance-like or NBS-LRR proteins, of which tae-miR2, tae-miR33, and tae-miR65 were detected only in one of the two cultivars (**Supplementary Table 11**).

**Figure 4:**
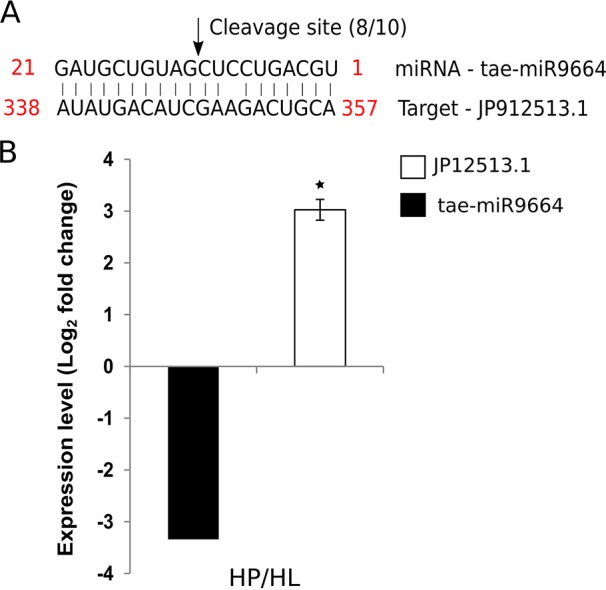
Target validation of tae-miR9664 and the expression of tae-miR9664 and its corresponding target gene. **(A)** Cleavage site validation for MIR9664 using 5’RLM-RACE. Eight out of 10 sequences of cloned products mapped to the cleavage site. **(B)** Expression levels of tae-miR9664 and target gene JP912513.1. Expression of JP12513.1 was determined with RT-qPCR and for tae-miR9664 from DESeq2 between healthy Penawawa vs Louise. * indicate P value significance of < 0.05.

The wheat miRNA tae-miR9664 is expressed in wheat seedlings, flag leaves, and developing seeds (Han et al., 2014). We predicted that tae-miR9664 targets the wheat transcript JP912513.1, which codes for a putative RPP13-like plant resistance protein of the nucleotide binding, leucine-rich repeat (NB-LRR) family. The predicted protein has a 29% amino acid identity to the barley Mla2 gene throughout most of its coding region. Modified 5’ RLM-RACE was performed to validate the cleavage of JP912513.1 by tae-miR9664. JP912513.1 was cleaved at the position predicted by psRNATarget, between 347 and 348 bps, in 8 out of 10 clones (**Figure 4A**). The other two cleavage products were at positions 370 and 371. To further validate the target, the expression of JP912513.1 mRNA was measured in flag leaf tissue in both Louise and Penawawa. The transcript abundance of the gene was 3.0-fold (log_2_ fold change) higher in healthy Penawawa (HP) compared with healthy Louise (HL) (**Figure 4B**). tae-miR9664 was downregulated -3.3-fold in healthy Penawawa vs. healthy Louise (**Supplementary Table 8**). The expression level of the transcript was opposite to that of tae-miR9664, providing evidence of miRNA-mediated regulation of JP912513.1. Alptekin et al. (2016) also suggested the role of tae-miR9664 in regulating kinase activity, providing further evidence for involvement of tae-miR9664 in a defense signal transduction pathway.

**Figure 5:**
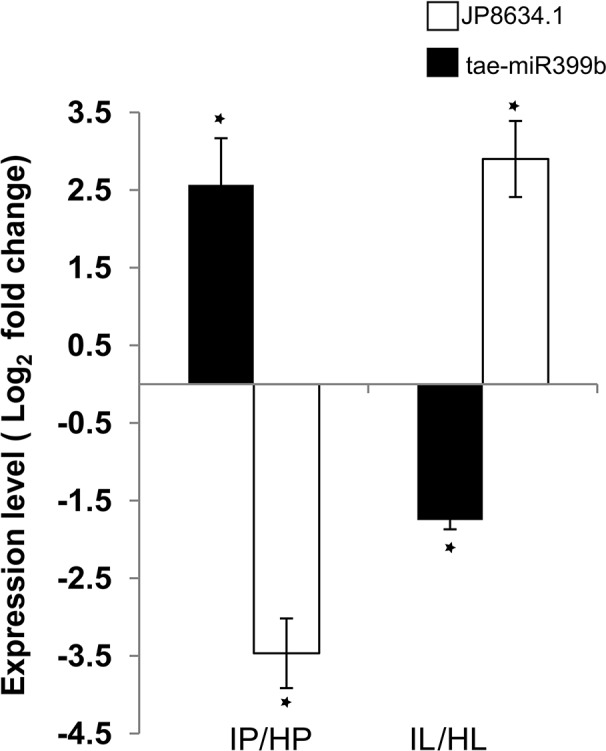
Expression levels of miR399b and target gene JP948634.1 measured by RT-qPCR. L and P refer to the cultivars Louise and Penawawa, respectively, and H and I refer to healthy and infected conditions. P-value significance of <0.05 is indicated with *.

TA078/miR399b showed cultivar-specific differential expression in response to infection. TA078/miR399b was expressed 2.6-fold higher in infected Penawawa than healthy Penawawa as measured by RT-qPCR: 1.7 fold increase in *in silico* analysis (**Figure 5**). The same microRNA was reciprocally downregulated -1.8-fold in Louise in response to infection (**Figure 5**). In *Arabidopsis*, miR399 targets a ubiquitin-conjugative enzyme (PHO2), which is involved in degradation of phosphate transporter proteins (Fujii et al., 2005; Bari et al., 2006; Lin et al., 2008). Since this target is conserved across plant species (Zhao et al., 2013), we tested whether gene expression of wheat ubiquitin-conjugating enzyme e2 24 (JP948634.1) varied with changes in expression levels of miR399b. Consistent with miRNA-directed regulation, a 2.6-fold increase in expression of miR399b in infected Penawawa correlated with a 3.5-fold decrease in JP948634.1 during infection, as measured by RT-qPCR. On the other hand, infected Louise flag leaves showed a -1.6-fold decrease in miR399b expression relative to healthy Louise, and accordingly 2.6-fold higher expression of JP948634.1 in infected Louise compared with healthy Louise (**Figure 5**). miR399-mediated gene regulation of a ubiquitin-conjugative enzyme is conserved in wheat and differs between two cultivars. Since miR399 is involved in regulation of phosphate transport, this also suggests phosphate starvation as a factor in wheat-*P. striiformis* interactions. Assessing the expression of other phosphate starvation induced genes, like IPS1, RNS1, PLDZ2 will be required to understand connection between phosphate starvation and rust infection. Further validation of target genes should also provide a better understanding of the genes regulated by wheat to resist stripe rust infection.

### Fungal Target Gene Prediction

Recent reports have provided evidence for cross-kingdom gene regulation during host–pathogen interactions (Wang et al., 2016; Zhang et al., 2016). When searched for fungal targets, 84 known miRNAs (including variants) and 66 novel miRNAs targeted *P. striiformis* transcripts (**Supplementary Tables 10** and **12**). Of these, tae-miR67 and tae-miR68 have more than 100 predicted targets from *P. striiformis* (**Supplementary Tables 11** and **12**). Four miRNAs, namely, tae-miR66, tae-miR67, tae-miR68, and tae-miR69, were exact matches to six *Pst* transcripts. We predicted a total of 469 *Pst* target genes, about 39.0% (183) of which were hypothetical proteins with no homology to known fungal proteins or associated GO terms. Since many effector proteins lack homology to annotated genes and are secreted, we predicted secretion signals for fungal proteins coded by these targets. A secretion signal was present in 25 of the known miRNA targets and 34 of the novel miRNA targets (**Supplementary Tables 13** and **14**). tae-miR38, expressed only in Louise, targets four fungal genes carrying a signal peptide, while tae-miR202 targets a 91 aa protein coding gene with four cysteine residues in its protein sequence. This suggests a possible role of wheat miRNAs in regulating effector genes in *Puccinia* through cross-kingdom RNAi. Previous studies in cotton show increased production of miR166 and miR159 that silence Ca^2+^-dependent cysteine protease (*Clp-1*) and an isotrichodermin C-15 hydroxylase (*HiC-15*), respectively, when infected with *Verticillium dahliae* (Zhang et al., 2016). This raises the possibility of conserved and non-conserved miRNAs evolving to target specific pathogen genes as a defense strategy against infection. Regulation of effectors by wheat miRNAs could contribute to cultivar-specific resistance as well. Additionally, such miRNAs can serve as suitable candidates for disease control *via* spray-induced gene silencing (Koch et al., 2016; Koch et al., 2018; Machado et al., 2017).

## Data Availability Statement

Publicly available datasets were analyzed in this study. This data can be found here: http://trace.ncbi.nlm.nih.gov/Traces/sra/sra.cgi?study=SRP060546.

## Author Contributions

NM, SR, and SH conceived the study. SH and NM provided the funding. PZ, NM, and SR performed the bioinformatic analysis. NM and SR performed the 5’RACE experiments. SR performed the RT-qPCRs. SR wrote the manuscript. SR, NM, and SH edited the manuscript.

## Funding

This work was supported by USDA National Institute of Food and Agriculture (NIFA) award number 2012-67013-19400 and Hatch project 1016563. NM is supported by a USDA National Institute of Food and Agriculture (NIFA) predoctoral fellowship 2017-67011-26066.

## Conflict of Interest

The authors declare that the research was conducted in the absence of any commercial or financial relationships that could be construed as a potential conflict of interest.
